# Persistent COVID an differential diagnosis with depression symptoms

**DOI:** 10.1192/j.eurpsy.2024.1040

**Published:** 2024-08-27

**Authors:** B. Arribas Simon, M. J. Mateos-Sexmero, O. Martin-Santiago, P. Andres-Olivera, B. Rodriguez-Rodriguez, P. Martinez-Gimeno, N. Navarro-Barriga, T. Jimenez-Aparicio, M. Andreo-Vidal

**Affiliations:** ^1^Psychiatry, Hospital Clinico Universitario, Valladolid; ^2^Psychiatry, Complejo Asistencial Universitario, Salamanca; ^3^Hospital Clinico Universitario, Valladolid, Spain

## Abstract

**Introduction:**

We present the case of a 48-year-old woman, a nurse, referred from the Internal Medicine department for evaluation of depressive symptoms and accompanying somatic presentation following COVID-19. The aim is to highlight a recently emerging condition that we are increasingly encountering in our clinics, which can complicate the diagnosis of an underlying affective disorder

**Objectives:**

Diagnosed with COVID-19, confirmed by a positive PCR test, 6 months ago following an infection in the workplace. The clinical picture consisted of mild symptoms, with a ten-day course and apparent resolution at the time of hospitalization. She returned to her work activities and gradually began to report fluctuating symptoms, including headaches, mild shortness of breath, fatigue, as well as a tingling sensation in the upper extremities, especially in the hands. Additionally, she described feelings of restlessness, depressive mood, and intense fatigue. In additional tests: (CT-Scan) there are signs of mild bilateral lower lung fibrosis.

**Methods:**

Treatment with Duloxetine was initiated for a case of depressive symptoms with accompanying physical symptoms. The differential diagnosis considered Major Depressive Disorder, Single Episode, and Adjustment Disorder with Depressed Mood.”

**Results:**

We are facing a clear case of depressive clinic that may have endogenous features, if we adhere to criteria such as those in the DSM-5, as it would meet the criteria for Major Depressive Disorder, Single Episode. However, we have a clearly identified trigger, so we also need to perform a differential diagnosis, primarily with Adjustment Disorder with Depressed Mood: here, the symptoms appear within 3 months following the stressful agent (in this case, SARS-CoV-2 infection). Unlike Major Depressive Episode, once the agent has ceased, the symptoms do not persist beyond 6 months (which we do not know because the physical symptoms causing disability have not disappeared).In addition to purely psychiatric diagnoses that we are accustomed to, we must consider a new diagnostic entity that is becoming more prevalent as the pandemic progresses, namely “long-covid” or persistent COVID.These are generally middle-aged women who, several months after infection, continue to manifest a multifactorial complex of symptoms. These symptoms persist over time, not only the classical ones but also many others that can appear during the ongoing course of the disease.

**Conclusions:**

Beyond the purely psychiatric diagnoses we are accustomed to, we must also consider a new diagnostic entity that is becoming more prevalent as the pandemic continues to advance: Persistent COVID or ‘long-COVID.’ Generally, this condition affects middle-aged women who, several months after contracting the virus, continue to exhibit a multifactorial complex of symptoms. The most common symptoms include fatigue/asthenia (95.91%); general discomfort (95.47%); headaches (86.53%); and low mood (86.21%)

**Disclosure of Interest:**

None Declared

